# Pulsed focused ultrasound enhances the therapeutic effect of mesenchymal stromal cell-derived extracellular vesicles in acute kidney injury

**DOI:** 10.1186/s13287-020-01922-1

**Published:** 2020-09-14

**Authors:** Mujib Ullah, Daniel D. Liu, Sravanthi Rai, Mehdi Razavi, Waldo Concepcion, Avnesh S. Thakor

**Affiliations:** Interventional Regenerative Medicine and Imaging Laboratory, Department of Radiology, Stanford University, Palo Alto, CA 94304 USA

**Keywords:** Extracellular vesicles, Mesenchymal stromal cells, Acute kidney injury, Focused ultrasound, Regenerative medicine, Homing

## Abstract

**Background:**

Acute kidney injury (AKI) is characterized by rapid failure of renal function and has no curative therapies. Mesenchymal stromal cell (MSC)-derived extracellular vesicles (EVs) are known to carry therapeutic factors, which have shown promise in regenerative medicine applications, including AKI. However, there remains an unmet need to optimize their therapeutic effect. One potential avenue of optimization lies in pulsed focused ultrasound (pFUS), where tissues-of-interest are treated with sound waves. pFUS has been shown to enhance MSC therapy via increased cell homing, but its effects on cell-free EV therapy remain largely unexplored.

**Methods:**

We combine pFUS pretreatment of the kidney with MSC-derived EV therapy in a mouse model of cisplatin-induced AKI.

**Results:**

EVs significantly improved kidney function, reduced injury markers, mediated increased proliferation, and reduced inflammation and apoptosis. While pFUS did not enhance EV homing to the kidney, the combined treatment resulted in a superior therapeutic effect compared to either treatment alone. We identified several molecular mechanisms underlying this synergistic therapeutic effect, including upregulation of proliferative signaling (MAPK/ERK, PI3K/Akt) and regenerative pathways (eNOS, SIRT3), as well as suppression of inflammation.

**Conclusion:**

Taken together, pFUS may be a strategy for enhancing the therapeutic efficacy of MSC-derived EV treatment for the treatment of AKI.

## Background

Acute kidney injury (AKI) is a condition characterized by a rapid deterioration of kidney function and is a common problem in hospitalized patients as well as those with comorbid chronic diseases [1]. In the past few decades, there has been a substantial increase in hospitalizations for AKI, with the Centers for Disease Control and Prevention (CDC) estimating the number hospitalizations in the USA increasing over fourfold from 953,926 in 2000 to 3,959,560 in 2014 [2]. Studies in other countries have shown similar trends, with dialysis-treated AKI reportedly increasing more than thirteen-fold in England from 1998 to 2013 [3] and approximately threefold in Denmark from 2000 to 2012 [4]. AKI is especially prevalent among critically ill patients and is often secondary to another pathology. In addition to being an independent risk factor for end-stage renal disease (ESRD) and death [5], multiple studies have shown that AKI may initiate the development of chronic kidney disease (CKD) or, where CKD is already present, accelerate its worsening [5–10]. Treatment for AKI is predominantly supportive, aimed at maintaining volume homeostasis and correcting biochemical abnormalities. Hence, there is an urgent need for new therapies that might actually repair injured kidneys and prevent the progression to worsening kidney disease.

Recently, there has been interest in the regenerative properties of mesenchymal stromal cell (MSC) therapy for the repair and regeneration of damaged kidney tissue [11, 12]. MSCs are a heterogeneous population of cells found in various adult tissues. Due to their immunomodulatory and regenerative properties, as well as their ease of isolation and in vitro expansion, MSCs have been extensively explored as a platform for cellular therapy [13]. Early animal studies have shown that MSCs are capable of homing to damaged kidney tissue and contributing to renal repair [14, 15]. Recently, it has become clear that the therapeutic effects of MSCs come not from their ability to directly differentiate into new cells, but rather by the release of soluble factors and extracellular vesicles (EVs) with regenerative properties [16]. These molecules act through paracrine signaling to stimulate repair via anti-inflammatory, mitogenic, vasculotropic, and pro-survival pathways, which provide protection for surviving intrinsic epithelial cells and promote their proliferation [17, 18]. Since MSCs act via paracrine signaling, their proximity to the injured site is believed to be crucial for tissue regeneration. However, multiple studies have shown that when MSCs are injected intravenously, they are predominantly trapped in the lung microvasculature, in what is termed the pulmonary first-pass effect [19–23]. Consequently, instead of using whole cells, many studies have now begun investigating the use of purified EVs from MSC as a cell-free therapy [24], which are small enough to avoid pulmonary trapping [25]. MSC-derived EVs carry a cargo of regenerative molecules and have been shown to have a therapeutic effect in various animal models of disease [26, 27].

Despite the growing interest in EVs for regenerative applications, there remains an unmet need to improve their therapeutic effect. One promising avenue of optimization could be the use of pulsed focused ultrasound (pFUS) [28]. pFUS uses short-duration, high-intensity pulses of sound waves to non-destructively sonicate target tissues. When combined with imaging guidance, pFUS can be targeted precisely to locations deep within the body. Previous studies have shown that pFUS can enhance MSC therapy for AKI [29, 30], but different mechanisms have been proposed for this effect. The homing hypothesis posits that sound waves transiently upregulate local inflammatory and chemoattractive signals [31–33]. This local gradient of cytokines, chemokines, and trophic factors has been shown to promote the homing of MSCs to various tissues including the skeletal muscle [34, 35] and the kidney [29, 30, 32, 36]. In the setting of a mouse model of cisplatin-induced AKI, this increased homing of MSCs after pFUS was able to improve renal function more so than MSCs alone [30]. However, we have previously demonstrated that pFUS can enhance MSC therapy without local upregulation of cytokines or increased MSC homing [29]. Instead, pFUS was shown to act through protein intermediates to spur endogenous proliferation pathways, an effect which synergized with MSCs. These two mechanisms may not necessarily be at odds with one another, but may instead reflect differences in ultrasound parameters leading to differences in subsequent bioeffects [28]. Although there have been many studies on the effect of pFUS on MSC therapy, little is known about its effect on EV therapy. Hence, in this study, we use a mouse model of cisplatin-induced AKI to assess the effect of pFUS on MSC-derived EV therapy.

## Methods

### Extracellular vesicle isolation, characterization, and purification

**Fig. 1:**
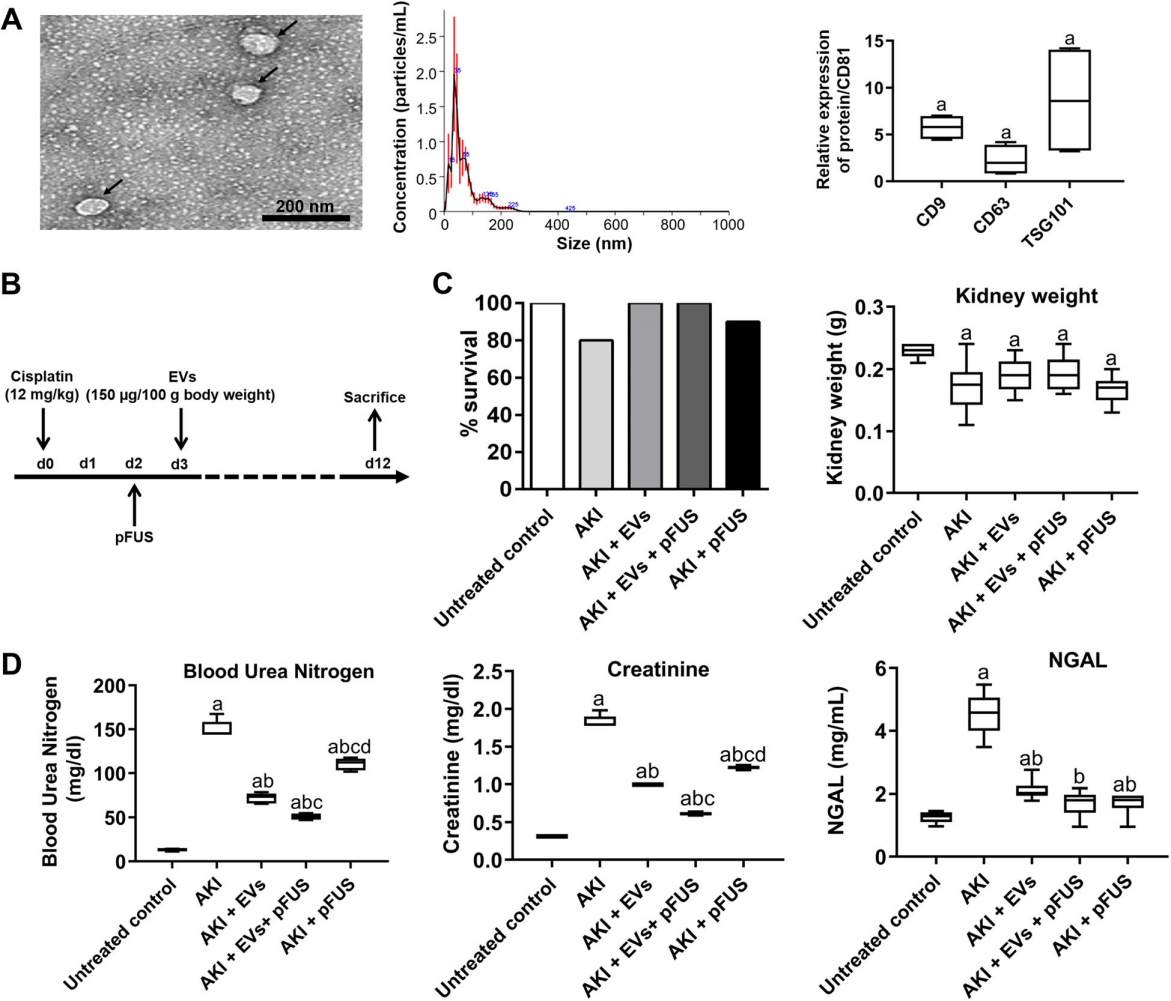
Physiological and biochemical parameters. **a** Characterization of EVs by transmission electron microscopy (left), size distribution measured by Nanosight tracking analysis (middle), and Western blot confirmation of EV markers CD9, CD63, and TSG101, normalized to CD81. Scale bar 200 nm. **b** Study protocol and treatment plan. **c** Survival rate at day 12 and kidney weight after cisplatin injection. **d** ELISA for blood urea nitrogen, creatinine, and NGAL level measured in blood plasma. Each group has *n* = 6 mice except for survival data, where *n* = 10. Significant difference ^a^*p* < 0.05: relative to untreated control; ^b^*p* < 0.05: relative to AKI; ^c^*p* < 0.05: relative to AKI-EV; ^d^*p* < 0.05: relative to AKI-EV-pFUS

Human bone marrow-derived MSCs (BM-MSCs) pooled from three donors were purchased from ATCC (PCS-500-012), transduced with GFP, and cultured in tissue culture flasks containing proliferative medium containing 10% fetal bovine serum (FBS), Dulbecco’s modified Eagle medium (DMEM), 100 μg/mL penicillin/streptomycin, and 2 mmol/mL glutamine (Thermo Fisher Scientific, USA) at 5% CO_2_ until passage 4, with culture media change every 2 days. The BM-MSCs were then plated in a flask at 2 × 10^4^ cells/cm^2^ with media containing DMEM, 100 μg/mL penicillin/streptomycin, 2 mmol/mL glutamine, and 3% EV-free FBS for the isolation of EVs. After overnight incubation, the conditioned media were centrifuged at 4 °C at 300×*g* for 10 min to remove dead cells, 17,000×*g* for 10 min to remove cellular debris, and 110,000×*g* for 90 min to pellet EVs. EVs were washed with PBS and ultracentrifuged again before use. We then applied this medium at room temperature to a column containing the anion exchange resin (Q Sepharose Fast Flow, GE Healthcare, IL, USA) balanced with 50 mM NaCl in 50 mM phosphate buffer and washed with 100 mM NaCl in 50 mM phosphate buffer and then rinsed with 500 mM NaCl in 50 mM phosphate buffer. EV fractions (20–30 mL) were pooled, dialyzed against PBS, filter sterilized, and used for this study. Nanoparticle tracking analysis (NTA) was performed using NanoSight NS300 (ATA Scientific, Australia) for assessment of the size and number of the isolated EVs. NTA 3.3 Software was used for measurements and data output. The mean diameter was 118 nm and standard deviation 27 nm, with sizes ranging from 20 to 180 nm (Fig. 1a). EVs were characterized by expression of the following human protein markers: CD9, CD63, CD81, and TSG101, all of which were positive.

### Animal experiments

All experimental procedures were performed in accordance with the guidelines and regulations of the Administrative Panel on Laboratory Animal Care (APLAC) at Stanford University. A total of 50 female CD1 mice (8 weeks old; body weight 30 ± 2 g) were purchased from Charles River Laboratories (Wilmington, MA, USA). All mice were housed for 1 week before any experiment in controlled conditions with 12-h light-dark cycles. EVs were isolated and purified by chromatography as previously described [37]. To investigate the protective effect of EVs and pFUS in cisplatin-induced AKI, animals were injected with cisplatin on day 0, administered pFUS on day 2, and treated with EVs on day 3 (Fig. 1b).

CD1 mice were randomly divided into 5 experimental groups, with each group containing 10 animals. Group 1 consisted of untreated control animals. For groups 2–5, animals received a single injection of cisplatin (12 mg/kg intraperitoneally) on day 0 to induce AKI. Group 2, AKI control, received no treatment; group 3, EVs alone, received EV treatment at a dose of 150 μg/100 g body weight via a tail vein injection on day 3; group 4, pFUS + EVs, received pFUS on day 2 followed by EVs on day 3; and group 5, pFUS alone, received pFUS only on day 2. At day 12 post-cisplatin injection, mice were sacrificed using an intraperitoneal injection of ketamine (100 mg/kg) and xylazine (10 mg/kg) followed by cervical dislocation, at which point blood and kidney samples were collected. For blood samples, the blood was collected to obtain serum which was then stored at − 20 °C for further analysis. One kidney was immediately immersed in 10% neutral buffered formalin for histological analysis while the other kidney was immediately frozen in liquid nitrogen for biochemical marker measurement and Western blot analysis. Subsequent analyses were conducted in a single-blind manner wherever possible.

### Pulsed focused ultrasound

A 1.1-MHz central frequency custom high-intensity focused ultrasound (HIFU) therapy transducer (H-102NRE, Sonic Concepts, Bothell, WA, USA) with 49-mm central opening was used. The HIFU transducer was calibrated in a water tank filled with degassed and deionized water. The transducer was driven by an Agilent 33250A function generator (Agilent Technologies, Santa Clara, CA, USA) and connected to a 50-dB ENI 525LA linear power amplifier (ENI Technology, Inc., Rochester, NY, USA) and an impedance matching circuit (Sonic Concepts, Bothell, WA, USA). The transducer was excited at central 1.1-MHz frequency with 20 cycles at 100-Hz pulse repetition frequency (PRF) in a “burst” mode. A hydrophone (HNR-0500, Onda Corporation, Sunnyvale, CA, USA) was placed in the focal spot of the transducer (55 mm away from its surface) and an Acoustic Intensity Measurement System (AIMS III, Onda Corporation, Sunnyvale, CA, USA) was used for precise movement and positioning of the hydrophone and digitizing the waveforms. The measured beam profile (full width half-maximum area for pressure) at the focal area was 10 mm long and 1.5 mm in diameter. The intensity and pressure measurements were performed for negative peak pressures (NPP) up to 3 MPa in order to reduce risks related to hydrophone damage. The obtained intensities and NPP values were then scaled to the desired PRF and duty cycle (DC) and linearly extrapolated to higher pressures/intensities.

For image-guided therapy to the mouse kidneys, a setup of co-aligned transducers was employed. The imaging transducer (Siemens Acuson S2000 14 L5 sp., Siemens Corporation, WA, USA) was placed in the central opening of the HIFU transducer. Both transducers were then aligned and fixed in a custom-made 3D-printed holder. The HIFU transducer’s focal spot was fixed at 55 mm axial, 0 mm lateral distance from the central point of the imaging transducer. Any misalignment of the HIFU and imaging beam was checked several times in the water tank with the hydrophone and oscilloscope by assembling and disassembling the 3D-printed holder. On all measurements, the measured beam misalignment was less than 200 μm. The ultrasound guidance of the in vivo kidney therapy was done with the Siemens S2000 scanner (Siemens Medical Solutions, Issaquah, WA, USA). Mice were kept under isoflurane anesthesia (2.5% induction, 0.1% maintenance) and submerged vertically in the water tank with the head kept above the water surface and body temperature maintained at 37 °C. The assembled holder with the HIFU and imaging transducers was connected to a translation stage and kept in the water at approximately 50 mm axial distance from the mouse. The mouse’s kidney was identified on the Siemens S2000 scanner and placed at the desired location, 55 mm axially and 0 mm laterally from the central point of the imaging transducer. To treat the whole kidney, 8 non-overlapping adjacent regions through the kidney were targeted for 30 s per region. The time to treat one kidney with these parameters was approximately 4 min. In order to deliver pFUS therapy to the animal, the HIFU transducer was used with the following parameters: 5% DC, 5 Hz PRF, 2.9 MPa PNP, and 272 W/cm^2^ spatial average pulse average intensity (*I*_SAPA_). After pFUS treatment, each mouse was removed from the water bath, dried, and placed in a recovery cage.

### Analysis of kidney function

Blood samples were collected retro-orbitally from anesthetized mice for analysis of biochemical levels, including blood urea nitrogen (BUN) and creatinine (Cr) using reagents purchased from Santa Cruz biotechnology USA according to the manufacturer’s instructions mentioned in the ELISA kit. Serum neutrophil gelatinase-associated lipocalin (NGAL) was measured using a mouse NGAL Quantikine ELISA Kit (R&D Systems, USA) according to the manufacturer’s instructions. The ELISA kit for kidney injury molecule-1 (KIM-1) and TIMP metallopeptidase inhibitor 1 (TIMP-1) were purchased from Cell signaling USA, samples were analyzed according to the manufacturer’s instructions, and results were measured and calculated at 450 nm via an ELISA reader (Bio-Rad, CA, USA) within 10 min. Urine samples were collected from both the control and AKI groups and also evaluated for KIM-1, TIMP-1, NGAL, and creatinine. Renal tissues were also homogenized, and the supernatant removed after 15 min of centrifugation at 3000×*g* at 4 °C. Next, NGAL and KIM-1 levels were measured using commercial kits according to the manufacturer’s instructions (R&D Systems, USA).

### Histology, immunohistochemistry, and immunofluorescence

At day 12, the mice were sacrificed and then perfused with 4% (vol/vol) paraformaldehyde in PBS to remove the blood from the tissues. Next, whole kidney tissues were fixed for 24 h in 4% (vol/vol) paraformaldehyde. After fixation and paraffin embedding, the kidney tissues were then sectioned into 6-μm slices for hematoxylin–eosin and trichrome staining. The slides were also stained with 4′,6-diamidino-2-phenylindole (DAPI) (D1306, Thermo Fisher Scientific, Santa Clara, CA, USA) and processed for GFP signal quantification using ImageJ software (NIH, USA) and flow cytometry as described previously [38].

### Pathological score for tubular injury

To determine kidney injury and rescue at the tissue level, we used a semi-quantitative histological scoring method previously defined by Klopfleisch et al. [39]. Injury was defined by dilated tubules, glomerular casts, tubular casts, necrosis, and tubular degeneration. The number of casts and tubular profiles showing necrosis were recorded in a single-blind fashion. Score 0 represents injury area less than 10%, whereas scores 1, 2, 3, and 4 represent injury involving 10–25%, 25–50%, 50–75%, or > 75% of the visualized field, respectively. At least 20 random fields at × 200 magnification were evaluated for each mouse with the average score calculated. For immunohistochemical staining, paraffin-embedded kidney sections were deparaffinized, hydrated, and antigen-retrieved, and endogenous peroxidase activity was quenched by 3% H_2_O_2_. Sections were then blocked with 10% normal donkey serum, followed by incubation with different antibodies such as interleukin 6 (IL-6) (M620, Thermo Fisher Scientific, Santa Clara, CA, USA) and tumor necrosis factor alpha (TNF-α) (M3TNFAI, Thermo Fisher Scientific, Santa Clara, CA, USA) overnight at 4 °C. After incubation with a secondary antibody for 1 h, sections were incubated with 3-3′-diaminobenzidine (DAB) (H-2200-30, Vector Laboratories, CA, USA). Slides were viewed with a microscope equipped with a digital camera (Nikon Eclipse 80i, USA) and then analyzed with a NanoZoomer (NanoZoomer S360Hamamatsu, USA).

### Western blot analysis

Kidney tissue was sliced into thin sections, followed by sonication and homogenization. The lysate was then placed in 1 × SDS sample buffer in association with radioimmunoprecipitation assay (RIPA) buffer solution containing 1% NP40, 0.1% SDS, 100 mg/ml PMSF, 1% protease inhibitor cocktail, and 1% phosphatase I and II inhibitor cocktail (Sigma, St Louis, MO, USA) on ice. The supernatant was then collected following centrifugation at 13,000×*g* at 4 °C for 30 min. Protein concentration was determined by a bicinchoninic acid protein assay. An equal amount of protein was loaded into 10% or 15% SDS-PAGE and transferred onto polyvinylidene difluoride membranes. The primary antibodies were used at 1:200 dilution unless otherwise noted: Akt (2920S, Cell Signaling Technology, MA, USA), p-Akt (PA5-95669, Thermo Fisher Scientific, Santa Clara, CA, USA), KIM-1 (PA5-98302, Thermo Fisher Scientific, Santa Clara, CA, USA), NGAL (ABS 043-29-02, Thermo Fisher Scientific, Santa Clara, CA, USA), TIMP-1 (MA5-13688, Thermo Fisher Scientific, Santa Clara, CA, USA), TNF-α (MM350C, Thermo Fisher Scientific, Santa Clara, CA, USA), NF-κB (PA5-16545, Thermo Fisher Scientific, Santa Clara, CA, USA), endothelial nitric oxide synthase (eNOS) (5880S, Cell Signaling Technology, MA, USA), SIRT3 (8469S, Cell Signaling Technology, MA, USA), PCNA (13-3900, Thermo Fisher Scientific, Santa Clara, CA, USA), VEGF (MA5-13182, Thermo Fisher Scientific, Santa Clara, CA, USA), survivin (PA1-16836, Thermo Fisher Scientific, Santa Clara, CA, USA), Bcl-2 (MA5-11757, Thermo Fisher Scientific, Santa Clara, CA, USA), BAX (MA5-14003, Thermo Fisher Scientific, Santa Clara, CA, USA), Caspase-3 (MA5-11521, Thermo Fisher Scientific, Santa Clara, CA, USA), p-ERK (MA5-15705, Thermo Fisher Scientific, Santa Clara, CA, USA), ERK (13-8600, Thermo Fisher Scientific, Santa Clara, CA, USA), p-MEK (sc-271914, Santa Cruz Biotechnology, TX, USA), MEK (sc6250, Santa Cruz Biotechnology, TX, USA), and anti-β-actin (sc-1616, 1:1000 dilution, Santa Cruz Biotechnology, TX, USA). Quantification was performed by measuring the intensity of the signals with the aid of the National Institutes of Health Image software package and Bio-Rad image software (Bio-Rad USA).

### Real-time PCR

RNA was extracted from kidney homogenized tissue using a Triazole Reagent (Sigma-Aldrich MO, USA). After digestion with DNase I, 2 μg RNA was reverse transcribed with the Applied Biosystems reverse transcriptase kit (Applied Biosystems, CA, USA). The quality of cDNA was assessed by the ratio of the absorbance at 260 nm and 280 nm using an Agilent 2100 Bioanalyzer (Agilent Bioanalyzer, CA, USA). cDNA was then amplified by PCR in an iCycler Thermal Cycler (Bio-Rad, CA, USA) with SYBR Green (Applied Biosystems, CA, USA) and specific primers for *Kim1* (Mm01291075_m1, Thermo Fisher Scientific, Santa Clara, CA, USA), *Timp1* (Mm01341361_m1, Thermo Fisher Scientific, Santa Clara, CA, USA), *Ngal* (Mm00443258_m1, Thermo Fisher Scientific, Santa Clara, CA, USA), *Il6* (Mm00446190_m1, Thermo Fisher Scientific, Santa Clara, CA, USA), *Tnfa* (Mm00443258_m1, Thermo Fisher Scientific, Santa Clara, CA, USA), *Nfkb1* (Mm00476361_m1, Thermo Fisher Scientific, Santa Clara, CA, USA), *Gapdh* (Mm99999915_g1, Thermo Fisher Scientific, Santa Clara, CA, USA), and *18S* (Mm02601777_g1, Thermo Fisher Scientific, Santa Clara, CA, USA); the latter two were used as housekeeping genes. The expression of marker genes was normalized to the endogenous *Gapdh* expression level and calculated with the 2^−ΔΔ*Ct*^ formula in % *Gapdh* expression [40].

### Statistical analysis

All data are expressed as the mean ± standard deviation (SD) and analyzed with analysis of variance (ANOVA) followed by the Tukey test. Statistical analysis was performed using the GraphPad Prism 8.0 software (Graphpad Software, Inc., San Diego, USA).

## Results

### EVs and pFUS reduce the clinical manifestations of AKI

EVs were confirmed to be positive for the expression of CD9, CD63, and TSG101, with quantification normalized to the internal control CD81 (CD9: 5.77 ± 1.34 relative expression; CD63: 2.25 ± 1.66 relative expression; TSG101: 8.64 ± 6.10 relative expression) (Fig. 1a). Cisplatin was administered to induce AKI on day 0, after which animals were treated with pFUS on day 2 or EVs on day 3 (Fig. 1b). There was a reduction in the survival of animals in the AKI (80%) and pFUS alone (90%) groups at day 12, compared to untreated controls (100%) (Fig. 1c). However, animals treated with EVs alone or pFUS + EVs showed no mortality at day 12. A similar trend was observed in kidney weight following cisplatin administration: compared to untreated controls, kidney weight was significantly reduced among animals in the AKI (0.17 ± 0.04 vs. 0.23 ± 0.01 g, *p* < 0.05) and pFUS alone groups (0.17 ± 0.02 vs. 0.23 ± 0.01 g, *p* < 0.05). Compared to the AKI group, treatment with EVs alone partially ameliorated this reduction in kidney weight (0.19 ± 0.03 vs. 0.17 ± 0.04 g, *p* > 0.05) (Fig. 1c). These trends were also reflected in serum laboratory values. At day 12, animals in the AKI group showed significantly elevated levels of several kidney injury markers compared to untreated controls, including blood urea nitrogen (BUN) (13.27 ± 1.26 vs. 150.23 ± 9.62 mg/dL, *p* < 0.05), creatinine (SCr) (0.31 ± 0.01 vs. 1.83 ± 0.08 mg/dL, *p* < 0.05), and NGAL (1.25 ± 0.19 vs. 4.54 ± 0.68 mg/mL, *p* < 0.05) (Fig. 1d). Compared to AKI, treatment with EVs alone significantly reduced the levels of all three injury markers (BUN 150.23 ± 9.26 vs. 72.23 ± 5.61 mg/dL, *p* < 0.05; SCr 1.83 ± 0.08 vs. 0.99 ± 0.01 mg/dL, *p* < 0.05; NGAL 4.53 ± 0.68 vs. 2.11 ± 0.33 mg/mL, *p* < 0.05), and the combined treatment of pFUS + EVs further reduced all these markers compared to treatment with EVs alone (BUN 50.74 ± 3.34 vs. 72.23 ± 5.61 mg/dL, *p* < 0.05; SCr 0.61 ± 0.02 vs. 0.99 ± 0.01 mg/dL, *p* < 0.05; NGAL 1.70 ± 0.42 vs. 2.11 ± 0.33 mg/mL, *p* > 0.05).

### EV homing to the kidney is not enhanced following pFUS

**Fig. 2:**
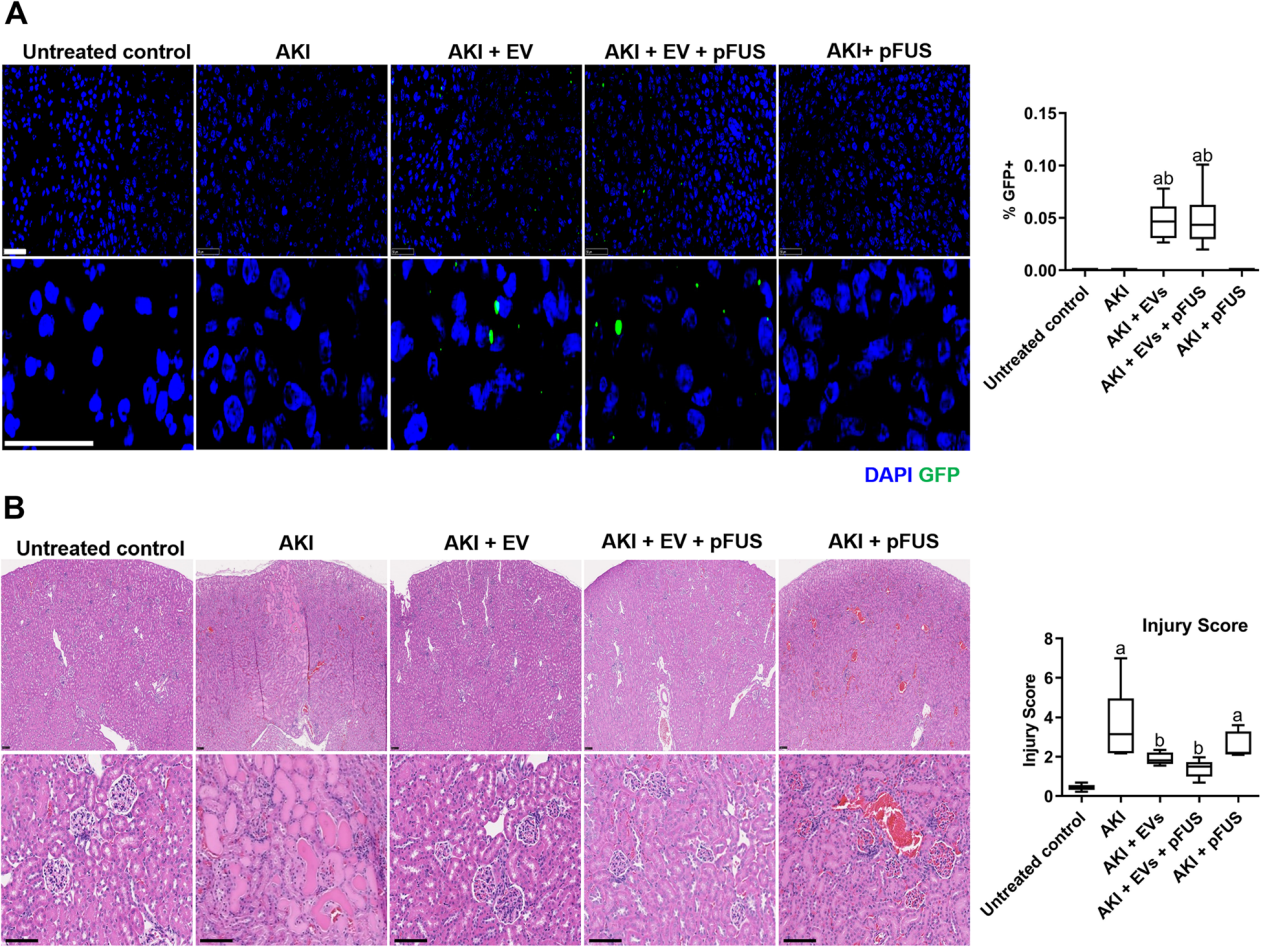
EV homing and kidney histology. **a** Homing of GFP-labeled EVs to the kidney, as measured by immunofluorescence (left) and flow cytometry in homogenized kidney samples (right). Scale bar represents 50 μm. **b** Hematoxylin and eosin staining of kidney sections, showing various levels of injury and cast formation (left), and quantification of injury score (right). Scale bar represents 100 μm. Each group has *n* = 6 mice. Significant difference ^a^*p* < 0.05: relative to untreated control; ^b^*p* < 0.05: relative to AKI; ^c^*p* < 0.05: relative to AKI-EV; ^d^*p* < 0.05: relative to AKI-EV-pFUS

Following the development of AKI in mice, IV-administered EVs were able to home to the injured kidney, as demonstrated by the detection of GFP-labeled EVs within the kidney tissue at day 12 (Fig. 2a). However, pFUS had no additional effect on EV homing, as demonstrated by both histological detection and flow cytometry analysis (0.05 ± 0.02% vs. 0.05 ± 0.03% GFP positive, *p* > 0.05). As expected, no expression of GFP was detected in the kidneys of animals not treated with EVs.

### EVs and pFUS reverse histological and molecular markers of kidney damage

Histological evaluation of kidney tissue demonstrated that cisplatin induced a significant increase in injury compared to untreated controls, as demonstrated by the presence of morphological changes within the kidney parenchyma, including glomerular casts, tubular casts, and fibrosis (Fig. 2b). This injury was reflected in a higher histological injury score in the AKI group compared to untreated controls (3.65 ± 1.81 vs. 0.43 ± 0.16, *p* < 0.05). Compared to the AKI group, those treated with EVs alone showed a dramatically lower injury score (3.65 ± 1.81 vs. 1.90 ± 0.31, *p* < 0.05). pFUS alone also resulted in a lower injury score (3.65 ± 1.81 vs. 2.57 ± 0.66, *p* > 0.05) that did not reach statistical significance. Animals in the combined pFUS + EVs group had further reduced injury scores compared to those receiving either EVs alone (1.40 ± 0.45 vs. 1.90 ± 0.31, *p* > 0.05) or pFUS alone (1.40 ± 0.45 vs. 2.57 ± 0.66, *p* > 0.05), though these differences did not reach statistical significance (Fig. 2b).

**Fig. 3:**
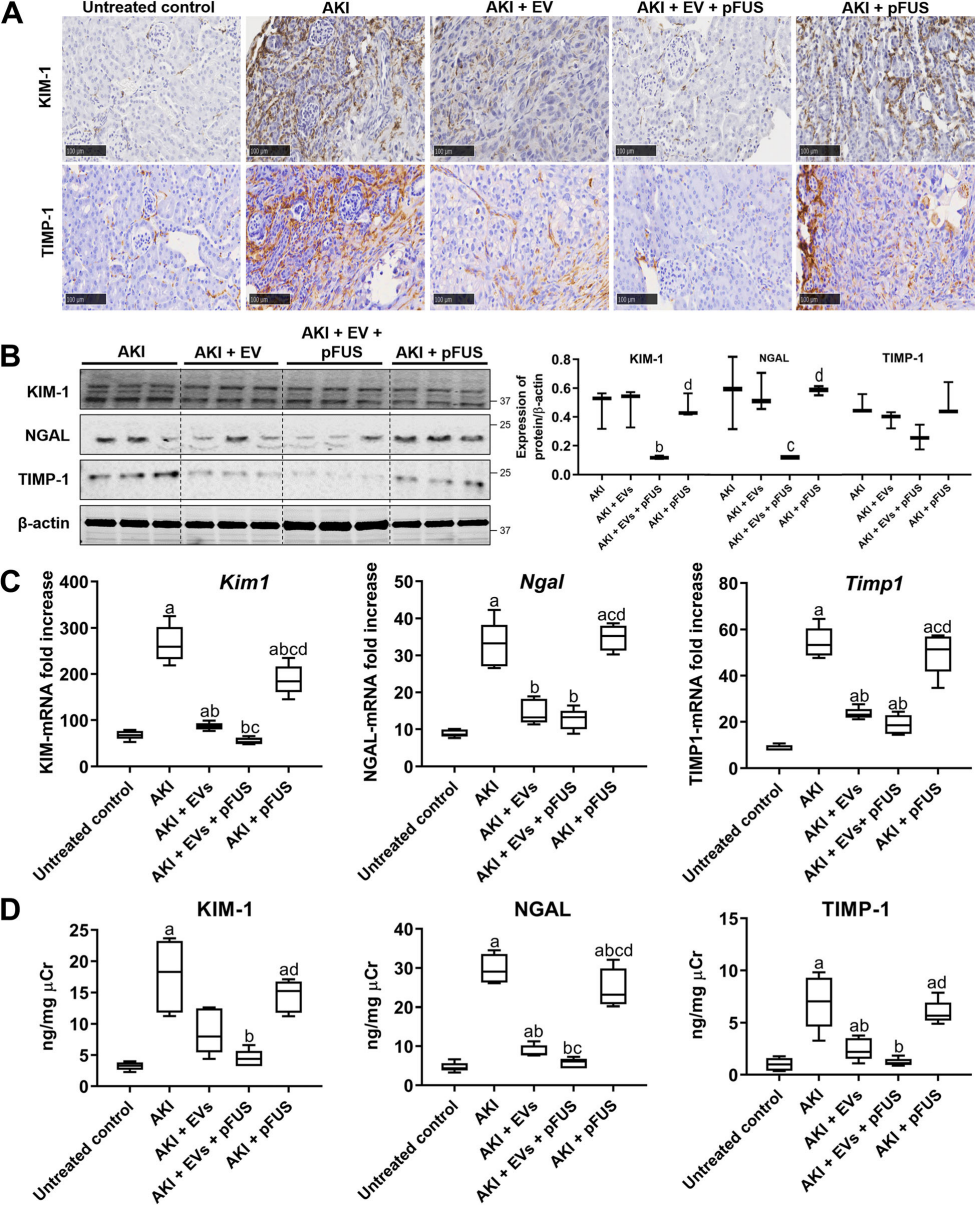
Molecular markers of kidney injury. **a** Immunohistochemical staining showing expression of injury markers KIM-1 and TIMP-1. Scale bar represents 100 μm. **b** Western blot for KIM-1, NGAL, TIMP-1, and β-actin (left), and their respective quantification (right). **c** Quantitative real-time PCR for *Kim1*, *Ngal*, and *Timp1* in the kidney tissue. **d** ELISA for KIM-1, NGAL, and TIMP-1 measured in the urine. Each group has *n* = 5 mice. Significant difference ^a^*p* < 0.05: relative to untreated control; ^b^*p* < 0.05: relative to AKI; ^c^*p* < 0.05: relative to AKI-EV; ^d^*p* < 0.05: relative to AKI-EV-pFUS

These trends were further confirmed by measuring the levels of tissue damage markers KIM-1 and TIMP-1. Immunohistochemical (IHC) staining of kidney tissue showed that untreated controls do not express these two molecules, but cisplatin-induced AKI strongly upregulated both markers (Fig. 3a). Although pFUS alone was not able to reduce their expression, EVs alone were able to partially reduce these two markers. Moreover, the combination of pFUS + EV treatment almost completely eliminated their presence (Fig. 3a). Western blot analysis further confirmed these trends: compared to the AKI group, EVs alone were able to partially reduce levels of KIM-1 (0.55 ± 0.16 vs. 0.38 ± 0.17 normalized expression, *p* > 0.05) and TIMP-1 (0.64 ± 0.40 vs. 0.38 ± 0.08 normalized expression, *p* > 0.05), but combined EVs + pFUS treatment showed much greater reductions in KIM-1 (0.55 ± 0.16 vs. 0.12 ± 0.02 normalized expression, *p* < 0.05), NGAL (0.44 ± 0.23 vs. 0.08 ± 0.02 normalized expression, *p* < 0.05), and TIMP-1 (0.64 ± 0.40 vs. 0.19 ± 0.11 normalized expression, *p* > 0.05) (Fig. 3b). qRT-PCR analysis on mRNA expression in kidney tissue (Fig. 3c), as well as ELISA analysis on urine samples, further recapitulated these trends (Fig. 3d).

### EVs and pFUS reduce inflammatory cytokines in the setting of AKI

**Fig. 4:**
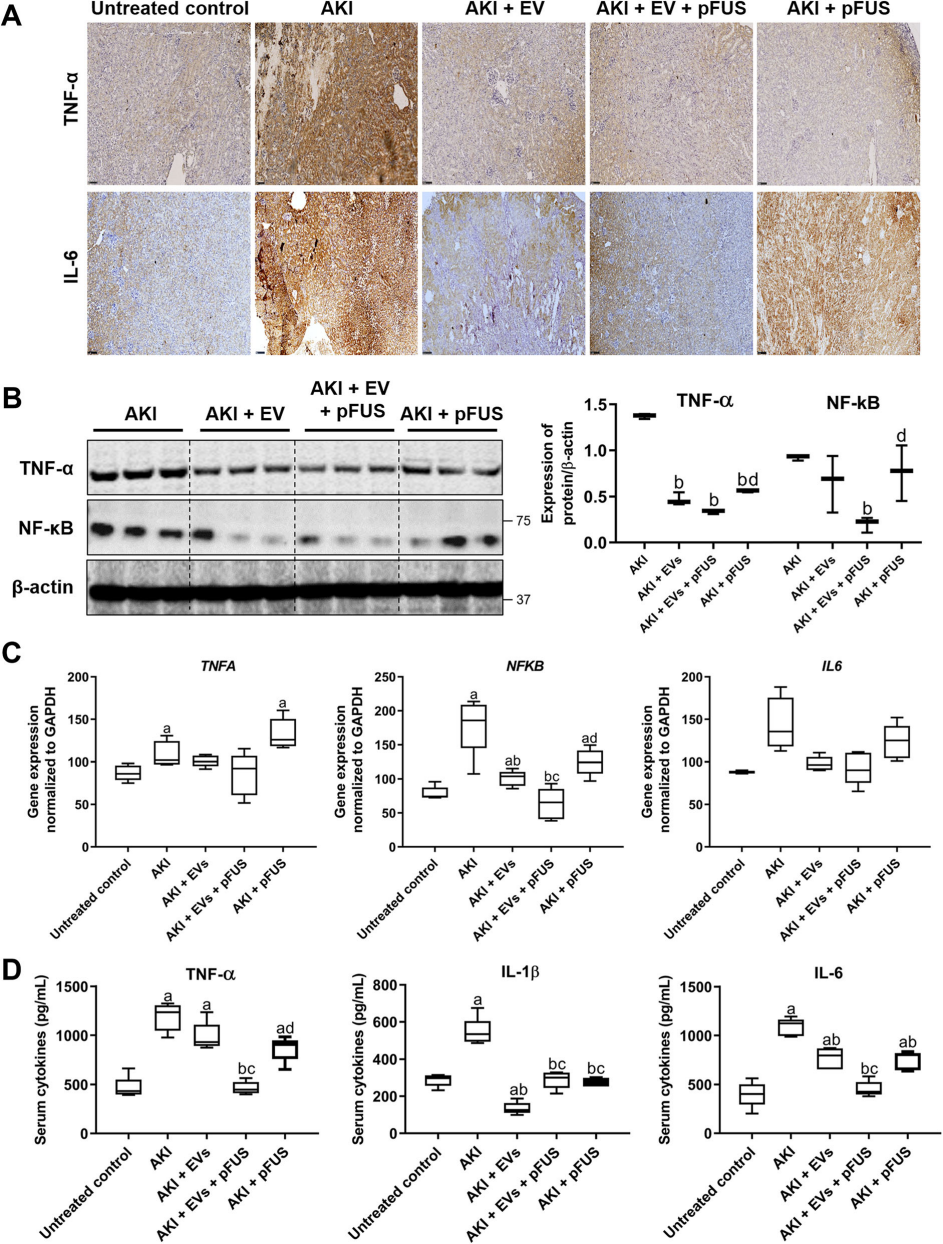
Inflammatory cytokines. **a** Immunohistochemistry staining for inflammatory markers TNF-α and IL-6 in kidney tissue. **b** Western blot on kidney tissue measuring inflammatory markers TNF-α and NF-κB (left), alongside their quantification (right). **c** Quantitative real-time PCR on kidney tissue measuring inflammatory markers *Nfkb*, *Il6*, and *Tnfa*. **d** ELISA measurement of blood serum concentrations of cytokines IL-1β, IL-6, and TNF-α. Each group has *n* = 5 mice. Significant difference ^a^*p* < 0.05: relative to untreated control; ^b^*p* < 0.05: relative to AKI; ^c^*p* < 0.05: relative to AKI-EV; ^d^*p* < 0.05: relative to AKI-EV-pFUS

Having established the regenerative capabilities of EVs, we next sought to elucidate the biological mechanisms through which they reverse AKI-induced kidney damage. We found by IHC that cisplatin-induced AKI is characterized by high levels of inflammatory markers in the kidney, including TNF-α and IL-6 (Fig. 4a). Treatment with either EVs, pFUS, or the combined treatment dramatically reduced the expression of these cytokines. Western blot analysis further supported these trends. Combined pFUS + EV treatment reduced TNF-α more so than EVs alone (0.34 ± 0.02 vs. 0.47 ± 0.07 normalized expression, *p* > 0.05), as was the case with its downstream signaling protein NF-κB (0.20 ± 0.08 vs. 0.65 ± 0.31 normalized expression, *p* > 0.05), though these results did not reach statistical significance (Fig. 4b). Similar trends were observed with qRT-PCR analysis of kidney tissue mRNA: compared to untreated controls, cisplatin-induced AKI resulted in elevated transcript levels of *Nfkb* (78.88 ± 9.85 vs. 178.76 ± 41.93 relative expression, *p* < 0.05), *Il6* (88.08 ± 1.65 vs. 144.56 ± 30.67 relative expression, *p* > 0.05), and *Tnfa* (87.07 ± 9.06 vs. 108.95 ± 14.96 relative expression, *p* < 0.05) (Fig. 4c). Treatment with EVs, with or without pFUS, was able to bring transcript levels of these cytokines back to levels comparable to those of untreated controls. ELISA analysis also showed a significant reduction in serum cytokine levels when comparing the AKI group with those treated with pFUS + EVs, including TNF-α (1187 ± 142 vs. 463 ± 65 pg/mL, *p* < 0.05), IL-6 (1087 ± 90 vs. 454 ± 78 pg/mL, *p* < 0.05), and IL-1β (547 ± 75 vs. 290 ± 47 pg/mL, *p* < 0.05) (Fig. 4d). In fact, animals treated with the combined pFUS + EVs showed significantly more potent reduction in IL-6 and TNF-α compared to those treated with EVs alone (IL-6 454 ± 78 vs. 769 ± 109 pg/mL, *p* < 0.05; TNF-α 463 ± 65 vs. 987 ± 143 pg/mL, *p* < 0.05; IL-1β 290 ± 47 vs. 135 ± 33 pg/mL, *p* < 0.05).

### EVs and pFUS promote proliferation and inhibit apoptosis

**Fig. 5:**
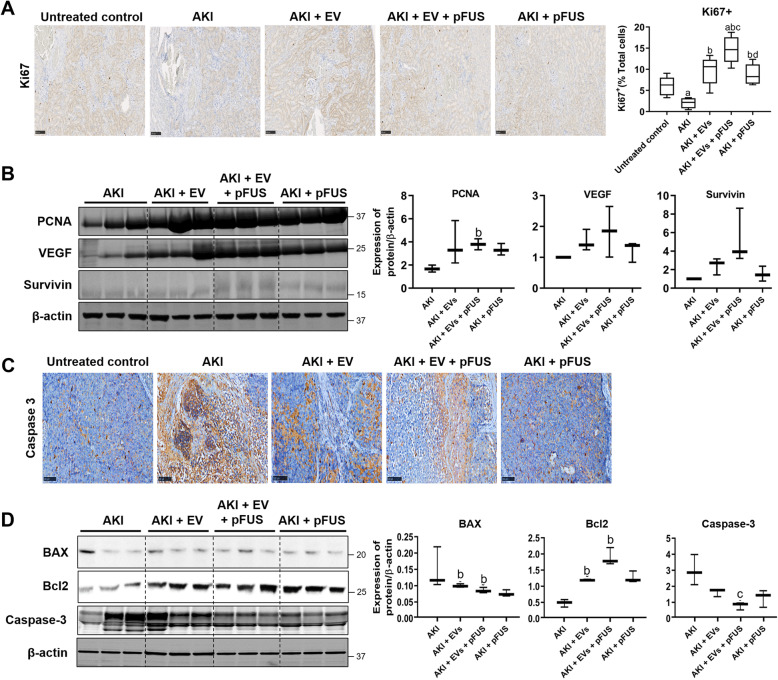
Proliferation and apoptosis. **a** Immunohistochemical staining for proliferation marker Ki67 in kidney tissue (left), alongside quantification of the percentage of Ki67-positive cells (right). **b** Western blot on kidney tissue measuring proliferation markers PCNA, VEGF, survivin, and β-actin (left) alongside quantification (right). **c** Immunohistochemical staining for apoptosis marker Caspase-3 in kidney tissue. **d** Western blot on kidney tissue measuring apoptosis markers BAX, Bcl-2, and Caspase-3 (left) alongside quantification (right). Each group has *n* = 3 mice. Significant difference ^a^*p* < 0.05: relative to untreated control; ^b^*p* < 0.05: relative to AKI; ^c^*p* < 0.05: relative to AKI-EV; ^d^*p* < 0.05: relative to AKI-EV-pFUS

Modulation of cell proliferation and apoptosis may be another mechanism by which EVs may exert their therapeutic effect. Using IHC staining, we found that compared to untreated controls, the AKI group had dramatically reduced cell proliferation in the kidney, as measured by the proliferation marker Ki67 (6.21 ± 2.26% vs. 2.01 ± 1.21% Ki67^+^, *p* < 0.05) (Fig. 5a). Compared to the AKI group, the percentage of proliferating cells was restored after treatment with EVs alone (9.7 ± 3.35% vs. 2.01 ± 1.21% Ki67^+^, *p* < 0.05) or pFUS alone (8.77 ± 2.45% vs. 2.01 ± 1.21% Ki67^+^, *p* < 0.05), and the combined pFUS + EV treatment showed the greatest effect (14.67 ± 3.20% vs. 2.01 ± 1.21% Ki67^+^, *p* < 0.05), significantly more than either treatment alone. We also measured the expression of several proliferation markers by Western blot, including PCNA, VEGF, and survivin. EVs alone were able to somewhat increase PCNA, VEGF, and survivin expression compared to the AKI group, though the difference did not reach statistical significance. pFUS alone was only able to increase PCNA levels. Compared to the AKI group, the combination of pFUS + EVs was able to increase the levels of PCNA (1.68 ± 0.30 vs. 3.80 ± 0.47 normalized expression, *p* < 0.05), VEGF (1.02 ± 0.46 vs. 1.85 ± 0.25 normalized expression, *p* > 0.05), and survivin (0.23 ± 0.04 vs. 1.16 ± 0.44 normalized expression, *p* > 0.05), more so than either treatment alone (Fig. 5b).

In addition to proliferation, we also measured apoptosis markers (BAX, Bcl-2, Caspase-3) in the kidney tissue. IHC analysis showed that AKI strongly upregulated Caspase-3, indicative of significant apoptosis following injury (Fig. 5c). Both EVs alone and pFUS alone reduced the amount of Caspase-3, but the combined treatment of pFUS + EVs showed an even greater reduction. Western blot analysis further showed that EVs alone strongly upregulated the expression of anti-apoptotic proteins like Bcl-2 compared to the AKI group (1.18 ± 0.02 vs. 0.47 ± 0.11 normalized expression, *p* < 0.05) and that the combination of pFUS + EVs resulted in an even greater increase compared to EVs alone (1.88 ± 0.27 vs. 1.18 ± 0.02 normalized expression, *p* > 0.05) (Fig. 5d). On the other hand, compared to the AKI group, pro-apoptotic genes like BAX and Caspase-3 were downregulated by both EVs alone (BAX 0.10 ± 0.01 vs. 0.15 ± 0.06 normalized expression, *p* < 0.05; Caspase-3 1.63 ± 0.24 vs. 2.99 ± 0.95 normalized expression, *p* > 0.05) and by pFUS alone (BAX 0.07 ± 0.01 vs. 0.15 ± 0.06 normalized expression, *p* > 0.05; Caspase-3 1.29 ± 0.53 vs. 2.99 ± 0.94 normalized expression, *p* > 0.05). The combination of pFUS + EVs resulted in a significantly stronger downregulation of Caspase-3 than either treatment with EVs alone (0.80 ± 0.23 vs. 1.63 ± 0.24 normalized expression, *p* < 0.05) or pFUS alone (0.80 ± 0.23 vs. 1.29 ± 0.53 normalized expression, *p* > 0.05).

### EVs and pFUS synergistically activate MAPK and Akt signaling

**Fig. 6:**
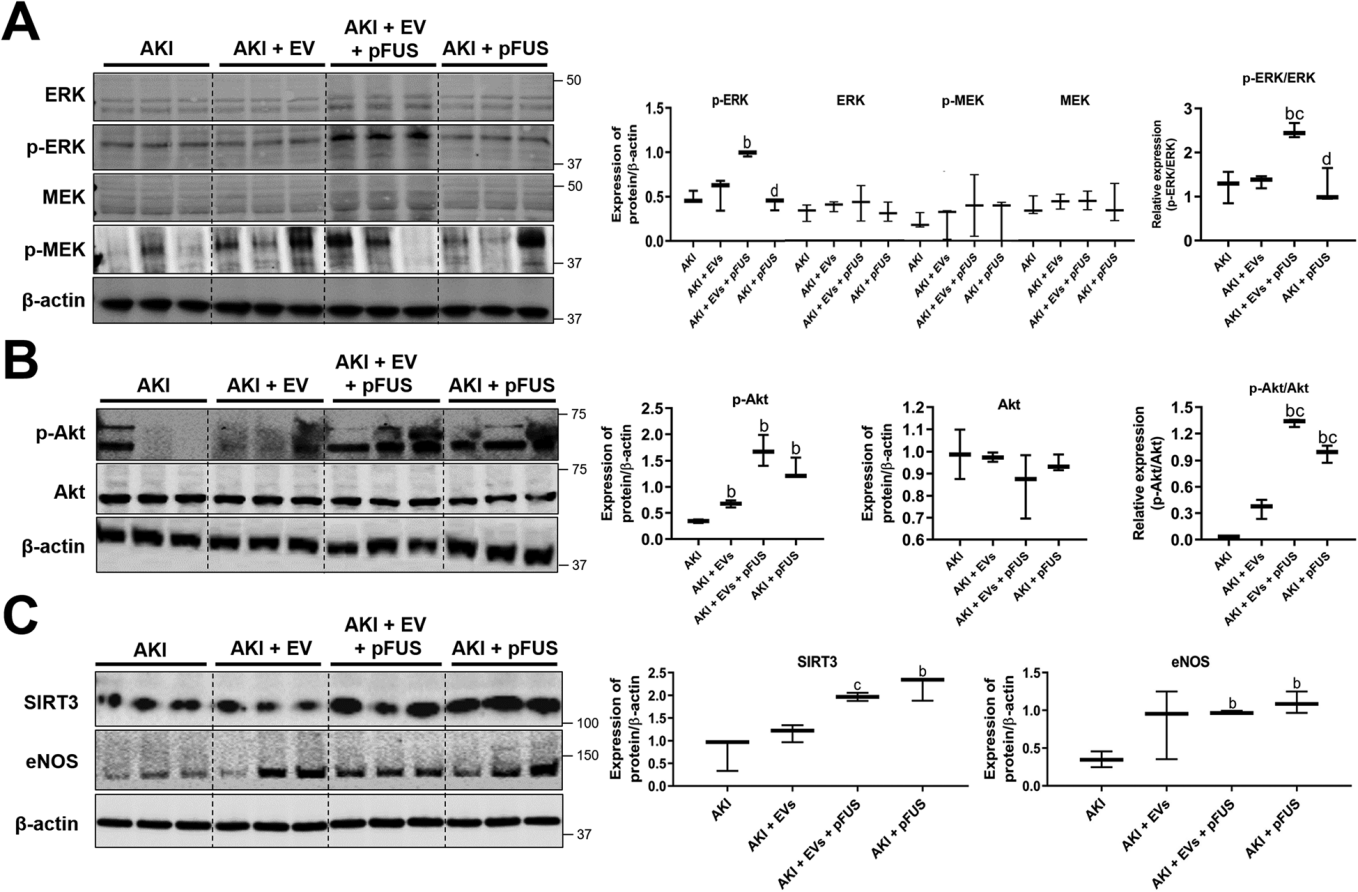
Signaling pathway analysis. **a** Western blot and protein quantification showing the expression of p-ERK, ERK, p-MEK, MEK, and β-actin in kidney tissue. **b** Western blot and protein quantification showing the expression of p-Akt, Akt, and β-actin in kidney tissue. **c** Western blot and protein quantification showing the expression of SIRT3, eNOS, and β-actin in kidney tissue. Each group has *n* = 3 mice. Significant difference ^a^*p* < 0.05: relative to untreated control; ^b^*p* < 0.05: relative to AKI; ^c^*p* < 0.05: relative to AKI-EV; ^d^*p* < 0.05: relative to AKI-EV-pFUS

We next sought to identify the molecular signaling pathways used by EVs to exert their regenerative properties. Western blot analysis of kidney tissue showed that compared to the AKI group, combined treatment with pFUS + EVs significantly upregulated MAPK/ERK signaling, as measured by the relative expression of phosphorylated to total ERK (1.23 ± 0.35 vs. 2.49 ± 0.16, *p* < 0.05) (Fig. 6a). However, no differences were found in the levels of phosphorylated or total MEK across the experimental groups. We also measured PI3K/Akt signaling based on the relative expression of phosphorylated to total Akt. Compared to the AKI group, PI3K/Akt signaling was upregulated by EVs alone (0.34 ± 0.03 vs. 0.68 ± 0.07, *p* < 0.05) and by pFUS alone (0.34 ± 0.03 vs. 1.32 ± 0.21, *p* < 0.05), with the combined treatment of pFUS + EVs resulting in an even greater upregulation (0.34 ± 0.03 vs. 1.69 ± 0.29, *p* < 0.05) (Fig. 6b). Finally, we measured the protein expression of SIRT3 and eNOS, two markers of regeneration and angiogenesis. Compared to the AKI group, SIRT3 was modestly upregulated by EVs alone (0.76 ± 0.37 vs. 1.18 ± 0.19 normalized expression, *p* > 0.05) and significantly upregulated by pFUS alone (0.76 ± 0.37 vs. 2.19 ± 0.27 normalized expression, *p* < 0.05). pFUS + EVs resulted in significantly higher upregulation of SIRT3 compared to EVs alone (1.96 ± 0.09 vs. 1.18 ± 0.19 normalized expression, *p* < 0.05). Compared to the AKI group, eNOS was upregulated in all three treatment groups: EVs alone (0.35 ± 0.11 vs. 0.97 ± 0.02 normalized expression, *p* < 0.05), pFUS alone (0.35 ± 0.11 vs. 1.12 ± 0.14 normalized expression, *p* < 0.05), and pFUS + EVs (0.35 ± 0.11 vs. 0.85 ± 0.46 normalized expression, *p* > 0.05) (Fig. 6c). However, in this case, the combined pFUS + EV treatment was not superior to the individual treatments.

## Discussion

In this study, we demonstrated that EVs are capable of reversing cisplatin-induced AKI and that this regenerative effect is enhanced when they are used in combination with pFUS. We have demonstrated several mechanisms by which EVs and pFUS exert a synergistic therapeutic effect, including (1) a reduction in inflammatory cytokines, (2) an increase in cell proliferation, and (3) a decrease in apoptosis. These effects are mediated by increased MAPK/ERK and PI3K/Akt signaling and upregulation of the SIRT3 and eNOS pathways.

pFUS presents a promising method for optimizing MSC-based therapies [28]. It is a non-invasive procedure that can be precisely targeted to deep structures in the body and has an excellent safety profile [32, 34]; indeed, focused ultrasound is already FDA-approved for various clinical applications [41]. Burks et al. have previously tested pFUS in the setting of cisplatin-induced AKI, though there are some discrepancies between their studies and ours [30]. In AKI, they found that pFUS alone did not significantly improve kidney function (creatinine and BUN), promote cell proliferation (Ki67 and p-Akt), or reduce apoptosis and necrosis. However, pFUS alone did elicit these effects in our current and previous experiments [29, 42]. This discrepancy may result from different ultrasound transducers and settings; given that there is a lack of data on how the biological effects of pFUS are influenced by specific ultrasound parameters, direct comparison between studies is difficult.

These discrepancies suggest that there are different molecular mechanisms by which pFUS operates, depending on ultrasound parameters. The studies by Burks et al. show that pFUS transiently activates TNF-α and IL-1α expression in the sonicated tissue, which act through NF-κB signaling to drive cyclooxygenase-2 (COX2) activity; in turn, this promotes the expression of several homing factors and increases the local accumulation of MSCs, thus augmenting their therapeutic effect [43, 44]. Our previous studies have shown, however, that pFUS can improve MSC therapy independently of improved homing [29]. Instead, pFUS modulates the expression of heat shock proteins (HSPs), leading to various downstream effects such as increased proliferation through PI3K/Akt signaling [29] and suppression of inflammation [42]. In line with these previously reported mechanisms, the current study has identified the involvement of MAPK/ERK and PI3K/Akt signaling, canonical pathways for cell proliferation and survival that play a role in facilitating regeneration of damaged tissue [45, 46]. Our study also implicates the SIRT3 and eNOS pathways. SIRT3 is a mitochondrial protein deacetylase known for its ability to regulate energy demand during stressful conditions, eliminate reactive oxygen species, and prevent apoptosis [47]. eNOS, the endothelial nitric oxide synthase, has been shown to play an important role in neovascularization [48]. The involvement of these two pathways further suggests that EVs and pFUS can induce angiogenesis and stimulate regeneration.

One notable result of our study is that pFUS did not increase EV homing to the kidney. Homing is not a passive process, but an active one that requires a sequential series of molecular interactions. The homing process for MSCs is well-characterized: in order to travel from circulation into a target tissue, they must undergo (1) tethering by selectins, (2) activation by chemokines, (3) arrest by integrins, (4) transmigration or diapedesis across the endothelial layer, and (5) extravascular migration to the target tissue mediated by cytokines [49]. pFUS at certain parameters can enhance this process by upregulating homing molecules at the target tissue, especially chemokines like SDF-1 and cell adhesion molecules like VCAM-1 [31–34]. Many studies have demonstrated increased MSC homing following pFUS [30, 32, 34, 35], but whether the same effect would be true of EVs was unknown. In our study, pFUS did not enhance EV homing. One possible explanation may lie in different pFUS parameters: different sonication intensities are known to elicit distinct biological effects, only some of which may generate the cytokine gradient responsible for enhanced homing [28, 50]. Another possible explanation is that the biology of EVs may not permit the same mechanisms of homing as their parent MSCs. In order for EVs to show enhanced homing following pFUS, they must express the relevant homing factors on their cell surface. EVs include particles that are generated within endosomal compartments and released when their surrounding compartment fuses with the cell membrane [26]. Hence, EVs would not necessarily have the same surface markers as their parent MSCs. However, they still express surface markers relevant for homing [51], such as the selectin receptor CD44 [52, 53] which facilitates initial tethering and rolling adhesion [54], and various integrins which facilitate homing to specific tissues [52, 53, 55]. More systematic efforts to characterize EV surface proteins would be valuable for assessing the feasibility of improving EV homing and reveal possible strategies for doing so. To our knowledge, there has only been one other study on the effect of pFUS on EV homing, though in a rather different setting. Bai et al. reported that pFUS increases the homing of blood serum-derived EVs to the brain by 4.5-fold [56]. However, several reasons may explain the discrepancy with our results. First, their EVs are derived from different sources and thus may express different homing factors. Second, the increased homing is likely more the result of blood–brain barrier opening, a known effect of pFUS on the brain [57], not true improvement of the molecular homing process. Finally, different ultrasound parameters could have elicited different biological effects between our studies.

Most previous studies attribute the enhanced therapeutic outcomes following pFUS to increased MSC homing. However, our results suggest there are other different mechanisms by which pFUS independently facilitates tissue regeneration. pFUS on its own seems to exert biological effects sufficient for tissue regeneration. Based on our data, pFUS alone was able to reduce inflammatory cytokines, induce PI3K/Akt signaling with subsequent increases in proliferating cells and decreased apoptosis, and upregulate SIRT3 and eNOS. Consistent with these findings, a previous study has shown that ultrasound can prevent AKI by stimulating cholinergic anti-inflammatory pathways [58], though that study used unfocused ultrasound. These effects of pFUS alone were observed in some previous studies [29, 42], but not others [30], which may be an effect of different ultrasound parameters. It is also possible that there may be some sensitization effect, wherein pFUS sensitizes the target tissue to the regenerative molecules carried by EVs, thereby increasing their therapeutic efficacy; this hypothesis, though exciting, requires further exploration.

## Conclusions

Our study demonstrates that the combination of pFUS + EVs is therapeutic in the setting of cisplatin-induced AKI, superior to either EVs or pFUS alone. AKI represents a growing clinical concern for which there are no curative therapies. Though there has been considerable preclinical success in using EVs therapeutically, more optimization remains to be done before they may reach clinical utility. EVs are an attractive cell-free alternative to MSC therapy. Their small size bypasses the pulmonary first-pass effect, where the vast majority of IV-infused MSCs become entrapped in the lung microvasculature [19–23]. EVs also obviate the need for a constant supply of cells and avoid concerns regarding unwanted engraftment or immune rejection [59]. Rigorous molecular characterization of the mechanisms underlying EV and pFUS therapy, alongside a better understanding of pFUS parameters, will be key to fully realizing their clinical potential.

## Data Availability

The data that support the findings of this study are available from the corresponding author upon reasonable request.

## Abbreviations

AKI: Acute kidney injury
BM: Bone marrow
BUN: Blood urea nitrogen
CKD: Chronic kidney disease
Cr: Creatinine
DAPI: 4′,6-Diamidino-2-phenylindole
DMEM: Dulbecco’s modified Eagle medium
eNOS: Endothelial nitric oxide synthase
ERK: Extracellular signal-regulated kinase
ESRD: End-stage renal disease
EV: Extracellular vesicle
GFP: Green fluorescent protein
HIFU: High-intensity focused ultrasound
IHC: Immunohistochemistry
IL: Interleukin
*I*_SAPA_: Spatial average pulse average intensity
KIM-1: Kidney injury molecule 1
MAPK: Mitogen-activated protein kinase
MSC: Mesenchymal stromal cell
NF-κB: Nuclear factor kappa-light-chain-enhancer of activated B cells
NGAL: Neutrophil gelatinase-associated lipocalin
NTA: Nanoparticle tracking analysis
PBS: Phosphate-buffered saline
PCNA: Proliferating cell nuclear antigen
pFUS: Pulsed focused ultrasound
PI3K: Phosphoinositide 3-kinase
RIPA: Radioimmunoprecipitation assay
SDF-1: Stromal cell-derived factor 1
SDS: Sodium dodecyl sulfate
SIRT3: Sirtuin 3
TIMP-1: TIMP metallopeptidase inhibitor 1
TNF-α: Tumor necrosis factor alpha
VCAM-1: Vascular cell adhesion protein 1
VEGF: Vascular endothelial growth factor
